# Use of Plant Extracts in Polymeric Scaffolds in the Regeneration of Mandibular Injuries

**DOI:** 10.3390/pharmaceutics16040491

**Published:** 2024-04-02

**Authors:** Bruna Eduarda Gandra de Oliveira, Fernanda Latorre Melgaço Maia, Lívia Contini Massimino, Claudio Fernandes Garcia, Ana Maria de Guzzi Plepis, Virgínia da Conceição Amaro Martins, Carlos Henrique Bertoni Reis, Vinícius Rodrigues Silva, Andre Alves Bezerra, Carolina Chen Pauris, Daniela Vieira Buchaim, Yggor Biloria e Silva, Rogerio Leone Buchaim, Marcelo Rodrigues da Cunha

**Affiliations:** 1 Orthopedics and Traumatology Sector, Faculty of Medicine of Jundiaí, Jundiaí 13202-550, Brazil; ra2001079@g.fmj.br (B.E.G.d.O.);; 2 Department of Implant Dentistry, Faculdade São Leopoldo Mandic, Campinas 13045-755, Brazil; fernanda@orallis.com.br; 3 Interunit Postgraduate Program in Bioengineering (EESC/FMRP/IQSC), University of São Paulo (USP), São Carlos 13566-590, Brazil; livia.cm@alumni.usp.br (L.C.M.); amplepis@iqsc.usp.br (A.M.d.G.P.); marcelocunha@g.fmj.br (M.R.d.C.); 4 São Carlos Institute of Chemistry, University of São Paulo, USP, São Carlos 13566-590, Brazil; claudiofgarcia@alumni.usp.br (C.F.G.); virginia@iqsc.usp.br (V.d.C.A.M.); 5 Postgraduate Program in Structural and Functional Interactions in Rehabilitation, University of Marilia (UNIMAR), Marília 17525-902, Brazil; dr.carloshenriquereis@usp.br (C.H.B.R.); danibuchaim@alumni.usp.br (D.V.B.); 6 Department of Biological Sciences, Bauru School of Dentistry, University of São Paulo (FOB/USP), Bauru 17012-901, Brazil; 7 Department of Human Anatomy, University of San Francisco (USF), Bragança Paulista 12916-900, Brazil; vinicius.rodrigues@usf.edu.br; 8 Postgraduate Program in Health Sciences, Faculty of Medicine of Jundiaí, Jundiaí 13202-550, Brazil; ra2303010@g.fmj.br (C.C.P.); ra2303011@g.fmj.br (Y.B.e.S.); 9 Graduate Program in Anatomy of Domestic and Wild Animals, Faculty of Veterinary Medicine and Animal Science (FMVZ), University of São Paulo (USP), São Paulo 05508-270, Brazil; 10 Medical School, University Center of Adamantina (UNIFAI), Adamantina 17800-000, Brazil

**Keywords:** collagen, elastin, hydroxyapatite, polymers, bone repair, bone regeneration, scaffolds, jatoba, pomegranate, plant extracts

## Abstract

Severe loss of bone mass may require grafting, and, among the alternatives available, there are natural biomaterials that can act as scaffolds for the cell growth necessary for tissue regeneration. Collagen and elastin polymers are a good alternative due to their biomimetic properties of bone tissue, and their characteristics can be improved with the addition of polysaccharides such as chitosan and bioactive compounds such as jatoba resin and pomegranate extract due to their antigenic actions. The aim of this experimental protocol was to evaluate bone neoformation in experimentally made defects in the mandible of rats using polymeric scaffolds with plant extracts added. Thirty rats were divided into group 1, with a mandibular defect filled with a clot from the lesion and no graft implant (G1-C, *n* = 10); group 2, filled with collagen/chitosan/jatoba resin scaffolds (G2-CCJ, *n* = 10); and group 3, with collagen/nanohydroxyapatite/elastin/pomegranate extract scaffolds (G3-CHER, *n* = 10). Six weeks after surgery, the animals were euthanized and samples from the surgical areas were submitted to macroscopic, radiological, histological, and morphometric analysis of the mandibular lesion repair process. The results showed no inflammatory infiltrates in the surgical area, indicating good acceptance of the scaffolds in the microenvironment of the host area. In the control group (G1), there was a predominance of reactive connective tissue, while in the grafted groups (G2 and G3), there was bone formation from the margins of the lesion, but it was still insufficient for total bone repair of the defect within the experimental period standardized in this study. The histomorphometric analysis showed that the mean percentage of bone volume formed in the surgical area of groups G1, G2, and G3 was 17.17 ± 2.68, 27.45 ± 1.65, and 34.07 ± 0.64 (mean ± standard deviation), respectively. It can be concluded that these scaffolds with plant extracts added can be a viable alternative for bone repair, as they are easily manipulated, have a low production cost, and stimulate the formation of new bone by osteoconduction.

## 1. Introduction

Reconstruction of the mandible is still a challenge for reconstructive surgery due to the complexity of the surgical procedure, which can range from simple rigid internal fixation to microvascular transfer of free tissue [1,2]. The surgical approach depends on the factors related to the mandibular lesion, such as trauma, infection, osteoradionecrosis, tumor removal, or ablative surgery of the oral cavity and lower face [3]. Due to this, some changes have become necessary in recent decades regarding the treatment of restoring the continuity of the mandibular segment in order to achieve the preservation of facial identity, the oral airway, speech, mastication, and functional maxillomandibular occlusion more effective [4].

Depending on the patient’s clinical conditions, there are several reconstructive options for mandibular defects, including 3D-printed PEEK implants [5], fibula bone–cutaneous flap [6,7], three-dimensional modeling of part of the scapula [8], titanium implant [9], non-vascularized autogenous bone graft [10], osteogenic distraction [11], microvascular tissue transfer [3], transfer using sternum and rib bone [12], free rib flap associated with muscles in the thoracic region [13], guided regeneration with non-resorbable membranes [14], gene therapies for bone reconstruction [15], and autologous grafts [16], which are considered the gold standard in bone reconstruction [17]. However, one of the problems with the use of autologous bone grafts is their early resorption [18], and this can jeopardize the long-term effectiveness of osseointegrated dental implants [19]. Thus, tissue-engineering strategies provide some alternatives to minimize these problems in the reconstruction of large segmental mandibular defects [20].

Based on this comparative information on mandibular continuity reconstruction methods, surgeons can choose a single technique or a combination of techniques, and associate them with alternative biomaterials that have demonstrated osteogenic capacity through experimental research in the field of tissue engineering [21,22,23,24]. These include natural polymers such as collagen, which mimic the extracellular matrix of bone and can also be combined with anti-inflammatory and antioxidant substances found in some plants [25]. Fruit extracts (grape seed, pomegranate peel, and jaboticaba peel, among others) have been used as cross-linking agents for collagen, and, given this, polymeric scaffolds with plant extracts added could be a promising alternative for bone reconstruction [26,27].

The scaffold serves as a temporary support for cell growth, aiding in the essential processes of cell proliferation, migration, and differentiation required for the formation of new tissue. However, for this to happen, the scaffold must be biocompatible, biodegradable, and non-toxic; have adequate porosity and a surface for cell adhesion; and be sterilizable [28,29]. Thus, among the materials with these characteristics, polymers of natural origin stand out due to their bioactivity, biocompatibility, and non-release of cytotoxic products during degradation [30,31]. In addition, the ability of natural polymers like elastin and collagen to support bone growth is crucial for the success of biomaterials, as they closely resemble the structure and properties of the organic bone matrix [32].

Elastin has been applied in conjunction with other components such as collagen, hydroxyapatite, bone morphogenetic protein (BMP), and chitosan in order to improve the structural properties of the biomaterial that are essential for cell proliferation [33,34,35,36]. Collagen is the most abundant protein in humans and is found in the extracellular matrix of connective tissues, providing support and protection for cells, as well as facilitating the supply of nutrients and oxygen [31]. Other biopolymers that are not present in the human body, such as a polysaccharide chitosan that can be obtained from *Loligo* sp., can be used in the development of new biomaterials [28].

However, when used in isolation, chitosan has certain limitations, such as water insolubility, poor mechanical strength, and weak antimicrobial properties [37,38,39]. Thus, the use of polymers associated with bioactive compounds can make scaffolds even more active, and among these substances, products extracted from natural plants such as jatoba that have anti-inflammatory action stand out [25,28,40].

Jatoba resin is rich in diterpenes and sesquiterpenes, two substances with potential biological activity [41]. This tree, whose scientific name is *Hymenaea courbaril*, is widely used in folk medicine due to its antimicrobial, antifungal, antibacterial, expectorant, laxative, and sedative properties [42]. In scaffolds, its presence does not alter the integrity of collagen fibers, and its concentration is directly related to increased roughness and hydrophobic character [28].

Pomegranate fruit has been used as a natural antibiotic to treat tonsillitis, pharyngitis, and other infections of the oropharyngeal cavity [43]. Pomegranate juice is a more powerful antioxidant than red wine [44] and is commonly used due to its phytoconstituents, such as tannins, polyphenols, alkaloids, flavonoids, anthocyanins, ascorbic acid, fatty acids, ursolic acid, and ellagic acid. Ellagic acid belongs to the group of hydrolysable tannins and has antioxidant, antiapoptotic, antimutagenic, antifibrosis, anti-inflammatory, antiatherosclerotic, antibacterial, and HIV-replicating properties. Ellagic acid acts on the proliferation of human bone marrow stem cells, as well as on the viability of osteoblasts and fibroblasts in vitro, in a dose-dependent manner [45,46].

From the context presented, it is admirable that there are countless possibilities for creating innovative scaffolds for bone repair. The properties of natural polymers used as a guide for bone regeneration [23,24] can be improved with the addition of other substances derived from vegetables and fruits, because the phytochemical compounds of some plants act to modulate bone signaling pathways [17]. Thus, studies into new combinations and concentrations of the natural substances that make up a scaffold are important for use in the treatment of craniomaxillofacial injuries, especially the mandible, in order to quickly restore the important function that this bone plays in the masticatory process, the voice, and the aesthetics of facial harmonization. Therefore, the aim of this study was to evaluate the osteogenic potential of natural polymers when combined with pomegranate extract and jatoba resin in the treatment of rat mandibular lesions.

## 2. Materials and Methods

### 2.1. Process for Obtaining Scaffolds

#### 2.1.1. Materials

Anionic collagen was extracted from bovine tendon and porcine serosa using an alkaline treatment, as described by Horn et al. (2015) [47]. Briefly, the bovine tendon was washed appropriately with a 0.9% sodium chloride (NaCl) solution to remove blood and other contaminants. It was treated with an alkaline solution (3 mL g^−1^ of tissue at 25 °C) containing hydroxides, chlorides, and sulfates of K^+^, Ca^2+^, and Na^+^ for 72 h. After, the tendon was passed through an aqueous solution with the same salts for 6 h. All salts were removed by washing in solutions of 3% boric acid, deionized water, and 0.3% ethylenediaminetetraacetic acid (EDTA). Using acetic acid (pH 3.5), the anionic collagen was extracted and kept under refrigeration (4 °C). Its concentration was determined by weighing dry mass (*n* = 3) and subsequently corrected to 1% with acetic acid (AcOH) [48].

Using water, the porcine serosa was cleaned, then cut and immersed in baths using sodium hydroxide solutions at a concentration of 0.5%, as well as acetic acid at 0.5%, aiming to remove fat, blood, and other debris. Next, alkaline hydrolysis was carried out using 3 mL g^−1^ of tissue at a temperature of 25 °C in a solution with hydroxides, chlorides, and sulfates of K^+^, Ca^2+^, and Na^+^. This process lasted 120 h. The hydroxides and salts were removed and the serosa passed through an aqueous solution with the same salts. The scaffolds were lyophilized and solubilized in AcOH solution (pH 3.5) until they reached a mass concentration of 4%. Before freeze-drying, all salts were removed by passing through solutions of 3% boric acid, deionized water, 0.3% EDTA, and deionized water again until a pH of 6.0 was achieved.

In order to remove blood and other contaminants, the bovine ear cartilage was cut (1 cm strips) and washed appropriately with 0.9% NaCl solution. Next, alkaline hydrolysis was carried out [47] following the same criteria as the collagen scaffold and changing the period, which was 15 h at a temperature of 37 °C. In AcOH solution of pH 3.5, the material was suspended, homogenized, and finally lyophilized.

Extracted from squid pens (*Loligo* sp.), as previously described by Horn et al. (2009) [49], chitosans were demineralized and deproteinized using diluted solutions of hydrochloric acid (HCl) and sodium hydroxide (NaOH) to isolate β-chitin. Deacetylation of N-acetyl groups was carried out using a concentrated sodium hydroxide solution (40% NaOH, *w*/*w*) at a temperature of 80 °C for a period of 3 h, using nitrogen as the atmosphere. The chitosan powder was obtained and solubilized in acetic acid of pH 3.5 to obtain a 1% chitosan gel (*w*/*w*). The degree of acetylation (11.2%) and molecular weight (1.6 × 10^5^ g mol^−1^) were determined by nuclear magnetic resonance (NMR) spectroscopy and capillary viscosimetry, respectively.

To determine the degree of acetylation, chitosan was dissolved in deuterated water (D_2_O) and deuterated hydrochloric acid (DCl). The analysis was carried out at 70 °C and 400 MHz frequency, accumulating a total of 64 scans on a nuclear magnetic resonance (NMR) spectrometer with Fourier transform, model 500/54 Premium Shielded (Agilent Technologies, Santa Clara, CA, USA). A sequence consisting of a delay of 6 s, a 90° pulse of 11 ms, and an acquisition time of 5.1 s was used. During the 6 s interval before the application of the 90° pulse, the solvent signal was suppressed by saturation using a long, low-power pulse at the solvent frequency. For the calculation, Equation $GA\;\left(\%\right)=\left(\frac{H_{Ac}}{3}/\frac{H_{2-6}}{6}\right)\times 100$ was used, which relates the integrals of the areas of the peaks referring to the methyl hydrogens in the acetylated groups and H_2−6._

Regarding the production of nanoHA, 100 mL of 0.01 mol L^−1^ of cetrimonium bromide (C_19_H_42_BrN) were added to 0.6 mol L^−1^ of potassium hydrogen phosphate (K_2_HPO_4_). After addition, the pH was adjusted to 12 with NaOH and the mixture was stirred for 2 h. Under constant stirring, 1.0 mol L^−1^ calcium chloride (CaCl_2_) solution was added to the previous solution, and the suspension formed was placed under reflux for 6 h and then ultrasonicated for 1 h. Therefore, the suspension was washed using deionized water and ethanol, with the precipitate placed at 40 °C for a period of 12 h in order to evaporate the solvent and then calcined at 550 °C for 5 h [50].

Jatoba resin was extracted from trees of the species *Hymenaea courbaril*. To purify the material, a solution of resin in ethanol in a ratio of 1:20 by mass was prepared and heated at 50 °C under stirring for 1 h in a closed flask. After cooling, it was filtered and dried under air flow, and a powder yield of 90.1% was obtained. A solution of purified jatoba resin in ethanol at a concentration of 160 mg mL^−1^ was prepared.

For the pomegranate peel extract (*Punica granatum*), fruit peels were washed, freeze-dried, and crushed to obtain a yellow powder. This powder was placed in a 60% (*v*/*v*) hydroethanolic solution, with stirring for 1 h at 50 °C [51]. Then, the suspension was filtered and the solvent was partially evaporated from the filtrate, which was then lyophilized to obtain the dry extract. Pomegranate peel extract solution was created in AcOH of pH 3.5 and ethanol (1:1) at a concentration of 25 mg mL^−1^.

#### 2.1.2. Scaffold Preparation

Two types of scaffolds were prepared. One of them was prepared with anionic bovine tendon collagen, chitosan, and jatoba resin (CCJ), and the other one with porcine serosa anionic collagen, nanohydroxyapatite, elastin, and pomegranate peel extract (CHER).

CCJ scaffold—A mixture of anionic collagen and chitosan (1:1 ratio) was prepared under mechanical stirring at 800 rpm (Fisaton^®^, mod. 715). One mL of ethanolic solution of jatoba resin was added to 50 g of the mixture and shaken for 2 h. Then, the mixture was placed in Teflon^®^ molds, frozen in liquid nitrogen, and lyophilized.

CHER scaffold—3.75 g of 4% collagen gel were diluted with 6.25 g of a suspension of 30 mg of nanoHA in AcOH of pH 3.5, yielding a collagen/nanoHA gel with a final concentration of 1.5% collagen. After, 50 mg of cartilage were added to 10 g of collagen/nanoHA gel. Sequentially, 1 mL of the pomegranate peel extract solution was dripped in 10 g of collagen/nanoHA/cartilage gel. Then, the mixture was placed in Teflon^®^ molds, frozen in liquid nitrogen, and lyophilized.

The scaffolds were neutralized in ammonia vapor (ammonium hydroxide 28.0–30.0% J.T. BAKER) for a period of 2 h and deaerated under air flow for at least 72 h.

### 2.2. Characterization Process of the Scaffolds

#### 2.2.1. Differential Scanning Calorimetry (DSC)

Differential scanning calorimetry was performed with DSC Mod 2010 equipment, TA Instruments. Twenty mg of the sample were placed in a hermetically sealed aluminum support under an N_2_ flow (80 mL min^−1^) and a heating ratio of 10 °C min^−1^ between 25 and 120 °C. The temperature of denaturation was given from the inflection point.

#### 2.2.2. Fourier Transform Infrared Spectroscopy (FT-IR)

CCJ gel and its precursors were diluted in acetic acid of pH 3.5 (1:3), placed in Teflon^®^ molds, and dried under air flow in order to form films by the casting method. The spectra were obtained in an FT-IR Shimadzu IR Affinity—1 in a 600 to 4000 cm^−1^ interval with 4 cm^−1^ of resolution and 32 scans. The CHER scaffold and its precursors were analyzed by FT-IR (ATR) (attenuated total reflectance) using a Bruker Tensor 27 FT-IR spectrophotometer in the range of 4000–600 cm^−1^, with a resolution of 2 cm^−1^ and 64 scans.

#### 2.2.3. Analysis of Scaffolds with Scanning Electron Microscopy (SEM)

The scaffold morphology of the surface and cross-section was analyzed by a SEM Zeiss Leo 440 (Cambridge, UK) with an Oxford detector (model 7060) operating with a 20 kV electron beam. The sample was covered with a thin layer of gold (BAL-TEC, Liechtenstein, Germany) with a 0.60 nm s^−1^ deposition rate, a chamber pressure of 2.00 × 10^−2^ mbar, and a 60 mA current. Using Martin’s diameter and measuring at least 30 pores, pore size was assessed with ImageJ^®^ software (1.48 version, Bethesda, MD, USA).

#### 2.2.4. Porosity

Porosity was performed following the procedure adapted from Garcia et al. (2021) [25]. The scaffolds were placed in a chamber in the presence of NaOH(s) for 24 h. Its dimensions were measured and the dry volume was calculated (V_1_). The scaffolds were weighed (W_1_) and placed in 5 mL of ethanol. After 24 h, the scaffolds were removed and weighed again (W_2_). The porosity was calculated according to Equation (1), with ρ_EtOH_ being the density of ethanol (0.790 mg mL^−1^). The procedure was performed in triplicate.

(1) $$\%porosity=\frac{\left(W_{1}-W_{2}\right)/\rho_{EtOH}}{V_{1}}\times 100$$

#### 2.2.5. Absorption in Phosphate Buffered Saline (PBS)

PBS (pH 7.4) absorption assays were performed at least in triplicate. The scaffolds were placed in a chamber in the presence of NaOH(s) for 24 h. The dried scaffolds were weighted, placed in PBS buffer, and reweighted at specific times after being gently dried on filter paper. The percentage of buffer absorbed was calculated using Equation (2).

(2) $$\%absorption=\frac{W_{wet}-W_{dry}}{W_{dry}}\times 100$$

In the equation, W_wet_ is the mass of the wetted scaffold at specific times, and W_dry_ is the initial mass of the dried scaffold.

### 2.3. Experimental Design

**Figure 1:**
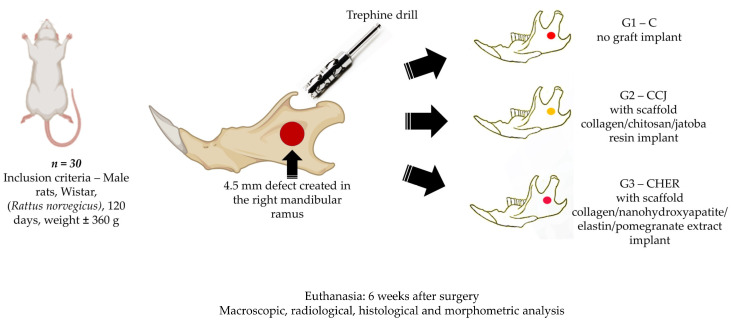
Experimental design. Inclusion criteria: Male Wistar rats (*Rattus norvegicus*) weighing approximately 360 g and 120 days old. Bone defect of 4.5 mm in the right ramus of the mandible. According to the filling of the bone defect, the animals were divided into three groups: group 1 (Control-C), *n* = 10, defect without graft, only clot; group 2 (CCJ), *n* = 10, defect filled with scaffold collagen/chitosan/jatoba resin; group 3 (CHER), *n* = 10, defect filled with scaffold collagen/nanohydroxyapatite/elastin/pomegranate extract. After 6 weeks of the experimental period, the animals were euthanized and samples from the surgical areas were submitted to macroscopic, radiological, histological, and morphometric analysis.

This study used 30 male rats (*Rattus norvegicus*, Wistar) aged 4 months, with an average weight of 360 g. They were kept in suitable conditions in the animal bioterium of the Faculty of Medicine of Jundiaí, Brazil. The project was approved by the Animal Experimentation Ethics Committee of the Jundiaí Medical School (CEUA/FMJ), protocol no. 172/21. The animals underwent a surgical procedure to experimentally create a 4.5 mm critical defect in the right ramus of the mandible and were divided into 3 groups of 10 animals each: group 1 with a mandibular defect filled with a clot from the lesion and no graft implant (control group), group 2 filled with collagen/chitosan/jatoba resin scaffolds (CCJ), and group 3 with collagen/nanohydroxyapatite/elastin/pomegranate extract scaffolds (CHER) (Figure 1).

### 2.4. Surgical Technique for Creating the Mandibular Defect

The rats were anaesthetized with Coopazine (xylazine) as a sedative, analgesic, and muscle relaxant and Dopalen (ketamine) as a general anesthetic, both of which were supplied by Kamimura (Trade in Products for Animals Ltd., Jundiaí, Brazil), in a 1:1 ratio and at a dose of 0.10 mg/100 g of body weight intramuscularly in the gluteal region. A total of 0.01 mg/100 g of body weight tramadol hydrochloride injectable solution was also applied subcutaneously to the back.

After anesthesia, the specimens were positioned in the left lateral decubitus position and the right side of the face was shaved, with subsequent asepsis of the area with 2% chlorhexidine digluconate solution. Next, an oblique incision was made in the skin in the middle third region, with the local musculature identified, which was sectioned and subsequently removed to expose the right ramus of the mandible. Using a trephine drill on a low-speed motor (Beltec LB–100^®^, Araraquara, Brazil), a 4.5 mm-diameter circular bone defect was created [52], which was filled as described in the experimental groups, except in group G1 (G-C), in which the bone defect remained filled only by the clot formed by the lesion. At the end of the surgical procedure, the soft tissues were repositioned and sutured. Each animal was isolated in its cage and given a special powdered feed (AIN 93 M Diet^®^, Isolated Soy Protein, Domeneghetti and Corrêa Ltd., Jaú, Brazil) that was mixed with filtered water to acquire a paste-like consistency in order to facilitate feeding the animals in the post-operative period. Water was also provided ad libitum. Post-operatively, the rats received a dose of 0.1 mg/100 g of weight of the antibiotic Pentabiotic (Fort Dodge^®^, Campinas, Brazil) daily for one week intramuscularly.

The animals were euthanized six weeks after surgery by intraperitoneal application of barbiturate thiopental at a dosage for rats (150 mg/kg): thiopental sodium 2.5% (Thiopentax^®^ Cristália, Itapira, Brazil), associated with the local anesthetic lidocaine hydrochloride (Xylestesin^®^ Cristália, Itapira, Brazil), at a dosage of 10 mg/kg. Once the animals had died, the mandibles were removed and photodocumented to analyze the clinical conditions of the operated area, followed by image analysis using digital radiography (AJEX-240 Diagnostic X-ray System^®^: 0.4–100 mA, 40–120 kVp, D-125 Inserted X-ray Tube and Pixx1717 apartment panel detector, Pixxogen). The samples were then immersed in formaldehyde solution and subjected to routine histological methods to make sections along the bone defect, and then stained with Masson’s trichrome to characterize bone neoformation and with Picrosirius red (saturated aqueous solution of picric acid plus 0.1 g of Sirius red F3B-Bayer^®^, St. Louis, MO, USA) to analyze the fibrillar components by polarized light microscopy [20,53,54].

The animals were randomized at the time of the experimental surgery and the microscopic analysis was blinded to the pathologists.

For quantitative assessment of the newly formed bone, we used the central region and an average distance between the peripheral and central regions. The measurements were transferred to the BioEstat 5.3 software [55] and ANOVA statistical tests with Tukey's post-test were applied, with a significance level of *p* < 0.05.

## 3. Results and Discussion

This study investigated a viable alternative to be used in dentistry and regenerative medicine for the treatment of mandibular lesions, given that this clinical condition has a direct impact on patients’ quality of life and on the resources allocated to healthcare for these purposes [56,57]. Therefore, the ability of plant extracts (pomegranate and jatoba) to stimulate osteogenesis in polymeric scaffolds during the bone repair process of defects created in the mandibular ramus of rats was evaluated. It is also noteworthy that the bioactive components of some plants constitute an alternative for tissue support in biological research and that their use in the manufacture of biocompatible and ecological biomaterials [58] contributes to reducing the use of animals and ethical concerns involved in experimental research [59,60].

This research proposal is also based on the use of natural macromolecules derived from plants that have applications in tissue engineering due to their easy accessibility, biocompatibility, support for cell proliferation, and extracellular matrix synthesis. In addition, the gel formation of these derivatives gives them the ability to create structures based on natural polymers that resemble extracellular matrices with indications for tissue regeneration [60]. In addition, the literature also demonstrates the osteogenic action of collagen and elastin scaffolds [23,34] and some phytochemical compounds derived from plants such as flavonoids, tannins, polyphenols, anthocyanins, terpenoids, polysaccharides, and alkaloids due to their properties as antioxidants, anti-inflammatories, and bone signaling pathway modulators [17]. The results obtained in this study were compared with data from other researchers, and some important differences and similarities were observed, but there were still some doubts about the ideal structure of biomaterials used in bone reconstruction, especially in the mandible. The following are the results and discussions, which are divided into a physical–chemical analysis of the scaffold and a clinical analysis of the graft in the experimental animals.

### 3.1. Physical–Chemical Analysis of the Scaffolds Used in the Experiment

**Figure 2:**
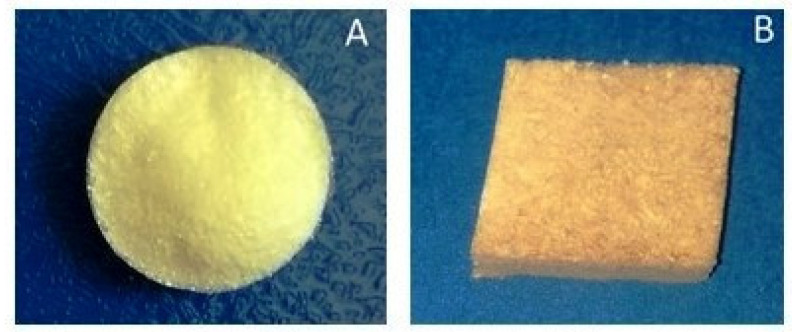
Digital photograph of scaffolds: (**A**) CCJ and (**B**) CHER.

The scaffold CCJ had a homogeneous appearance with a yellowish color due to the presence of jatoba resin. CHER was brownish due to the presence of pomegranate peel extract (Figure 2). The collagen present in CCJ scaffold showed a denaturation temperature of the 45.9 °C, while CHER showed a value of 47.7 °C.

**Figure 3:**
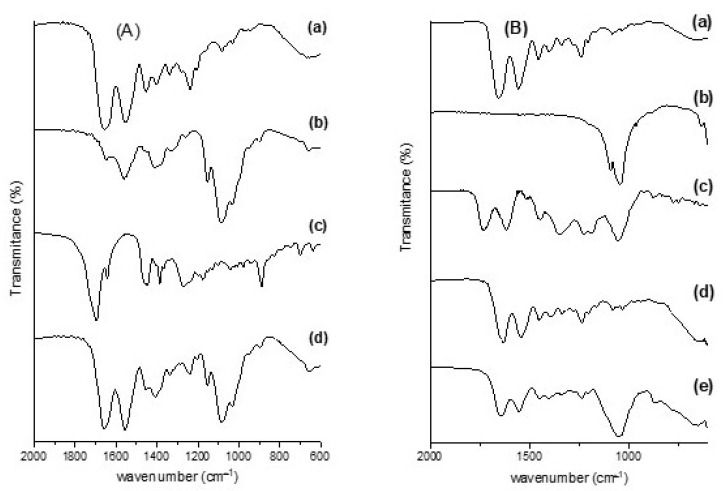
FT-IR spectra of (**A**) CCJ scaffold: (**a**) collagen; (**b**) chitosan; (**c**) jatoba resin; (**d**) collagen/chitosan/Jatoba resin. (**B**) CHER scaffold: (**a**) collagen; (**b**) nano-hydroxyapatite; (**c**) cartilage; (**d**) pomegranate peel extract; (**e**) collagen/nanoHA/cartilage/pomegranate peel extract.

FT-IR spectra of the scaffolds and the precursors are shown in Figure 3. Characteristic bands of anionic collagen (Figure 3(Aa,Ba)) were observed in the region of 3320–3330 cm^−1^, referring to the O-H and N-H deformations; at 1656–1659 cm^−1^, characteristic of axial deformation of C=O and C-N bonds (amide I); in 1558 cm^−1^ of angular deformations of NH bonds (amide II); in the region of 1454–1456 cm^−1^, referring to the pyrrolidine ring; and in 1236–1238 cm^−1^ of amide III [61,62].

The bands related to chitosan (Figure 3(Ab)) were seen at 1651 cm^−1^ for deformation C=O (amide I); at 1560 cm^−1^, corresponding to N–H deformation (amide II); at 1415 cm^−1^ due the deformation of the C–H bond; and at 1084 cm^−1^, referring to the C–O stretching characteristic of the saccharide structure [63,64].

In the FT-IR spectrum of jatoba resin (Figure 3(Ac)), bands referring to the C–H bonds of methyl groups were observed at 2931, 2866, 1449, and 1387 cm^−1^; the double bonds C=C and C=O were indicated by bands at 1697 and 1641 cm^−1^; the bands observed at 1175 and 1267 cm^−1^ refer to the presence of esterified succinic acid; and the narrow band at 887 cm^−1^ refers to exocyclic methylene groups [65]. In the spectrum of the collagen/chitosan/jatoba resin scaffold (Figure 3(Ad)), it was possible to observe an increase in the intensity of the band only in the region of 2945 cm^−1^ of methyl groups.

The FT-IR spectrum of nano-hydroxyapatite (Figure 3(Bb)) showed the stretching of the O‒H group close to 3200 cm^−1^ and the stretching and deformation of the phosphate ion (PO4)^3−^. The bands referring to the phosphate ion were observed in a pronounced way at 1083 and 1037 cm^−1^, referring to the asymmetric stretching; there was an attenuated band at 957 cm^−1^, referring to the symmetric stretching of the phosphate; and there were three bands at 605, 567, and 464 cm^−1^, referring to asymmetric deformation [66,67,68].

Auricular cartilage showed bands similar to collagen in its spectrum due to its composition (Figure 3(Bc)) [69]. The spectrum of pomegranate peel extract (Figure 3(Bd)) showed a band at 3400 cm^−1^, resulting from the O‒H stretching present in the hydroxyls of flavonoids. The band at 1728 cm^−1^ was attributed to C=O stretching of carboxylic groups or C=N. The band at 1615 cm^−1^, accompanied by others of lower intensity close to 1514 cm^−1^, corresponded to the stretching of the C=C‒C aromatic ring. The band at 1449 cm^−1^ referred to the stretching of the C=C groups of the aromatic ring. The band at 1045 cm^−1^ occurred due to deformation of the C‒H groups of the aromatic ring and O‒H groups [70,71].

In the case of the CHER scaffold (Figure 3(Be)), the presence of proteins was observed by the presence of amine bands I, II, and III at 1643, 1554, and 1237 cm^−1^, respectively. Nano-HA was characterized by an intense band at 1046 cm^−1^ due to phosphate ions. The presence of pomegranate peel extract was not be observed.

**Figure 4:**
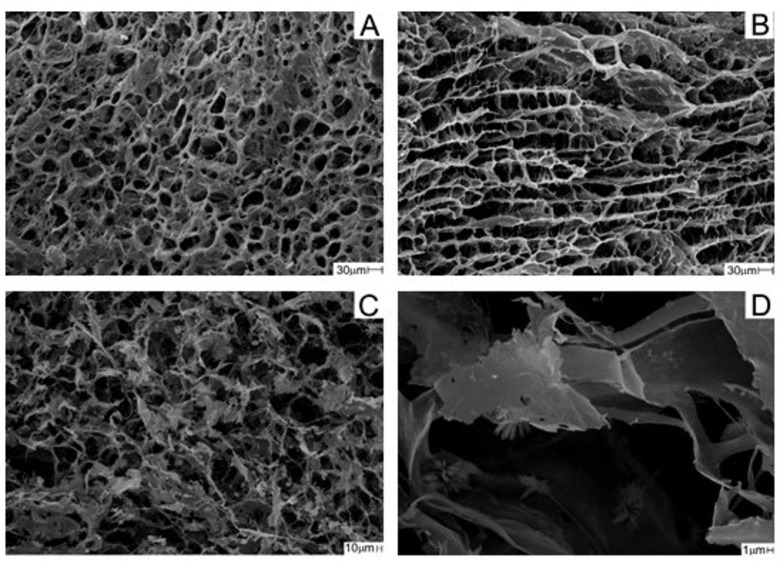
SEM micrographs of the scaffolds: (**A**) superficial CCJ, (**B**) transversal section CCJ, and (**C**) superficial CHER, all with 500x magnification, and (**D**) superficial CHER 6000× magnification.

SEM micrographs showed an open porous structure in both scaffolds with a homogeneous distribution (Figure 4A,C). A uniformly interconnected network was observed in the internal structure of both scaffolds, as exemplified by the CCJ scaffold (Figure 4B). The presence of nanohydroxyapatite in CHER scaffold was observed at 6000× magnification (Figure 4D).

**Figure 5:**
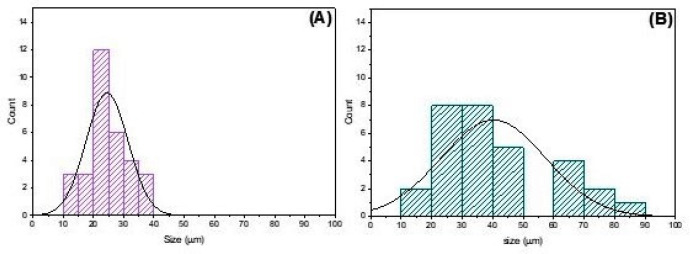
Pore size distribution histogram for scaffolds (**A**) CCJ and (**B**) CHER.

The pore size distribution in the scaffolds is shown in Figure 5, with an average pore size of 24 ± 7 µm for CCJ and 40 ± 17 µm for CHER. A greater homogeneity in pore distribution was observed for CCJ than for CHER. However, in the latter case, there was the presence of larger pore sizes (Figure 5). The porosity scaffolds were very similar: CCJ had a porosity of 83.6 ± 3.1%, while for CHER it was 86.7 ± 5.1%.

**Figure 6:**
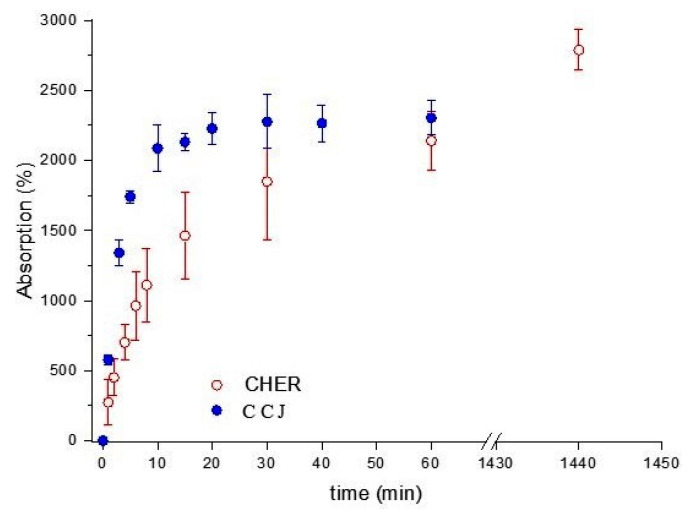
Absorption degree of scaffolds (-●-) CCJ and (-○-) CHER.

The degree of absorption of the matrices is shown in Figure 6. CCJ had a maximum absorption of 2300% at 60 min, while CHER had a maximum absorption of 3400% at 1440 min. Observing the behavior of both scaffolds at 30 min, an absorption of 2277 ± 192% for CCJ and 1849 ± 414% for CHER was obtained; that is, the absorption for the CHER matrix was greater but its swelling was slower than for CCJ.

The collagen used in the development of the scaffolds had a greater amount of negative charge due to the selective hydrolysis of carboxyamide groups, thus increasing biocompatibility [72,73]. DSC was performed to verify the integrity of the collagen triple helix. The CCJ scaffold presented a slightly lower Td (45.9 °C) than the anionic collagen scaffold (47.9 °C) obtained by Massimino et al. (2020) [74]. This decrease was due to the presence of jatoba resin, as well as other natural additives, which, when linked to the polymer chain, alter its structure and consequently its Td. The CHER scaffold presented a higher Td (47.7 °C) due to the presence of pomegranate extract. Garcia et al. (2021) [25] obtained porcine collagen scaffolds with different natural extracts and observed a significant increase in Td.

In the FT-IR spectrum of the scaffolds (Figure 3), the characteristic bands of the precursors were observed, in agreement with other studies [25,75]. The values of the characteristic bands for collagen in the infrared region were directly linked to its extraction source, with only small displacements observed [76]. According to Tappert et al. (2013) [65], a band in the region of 1265 cm^−1^ indicates a recently collected resin, while a band at 1235 cm^−1^ indicates the formation of amber. In this FT-IR spectrum of jatoba resin (Figure 3(Ac)), an intense band was observed at 1267 cm^−1^, which allows us to say that the resin used was recently collected. However, in the spectrum of the CCJ scaffold, it was possible to observe an increase in the intensity of the band only in the region of 2945 cm^−1^, since the other characteristic bands of the resin overlapped those of collagen and chitosan. For the CHER scaffold, the presence of collagen and cartilage was observed through amine bands I, II, and III and nano-HA through the intense band of phosphate ions; however, the bands referring to the pomegranate peel extract were overlapped by those of the proteins.

CCJ and CHER scaffolds presented suitable morphology for use in bone regeneration, with superficial pores (24 ± 7 and 40 ± 17 µm, respectively) and interconnected channels. Several factors can influence the cell growth process, one of which is the size of the pores. For neovascularization to occur 5 µm are required, while for the growth of osteoid tissue 75–100 µm are required [77]. Penetration of fibrous tissue requires pores of 10–75 µm, and for bone regeneration they must be 100–350 µm [78]. Therefore, the matrices obtained in this study had pores that would be suitable for being used in different ways for cell growth.

According to Iacob et al. (2018) [79], fluid absorption, as well as the colonization rate and angiogenesis process, are directly influenced by the percentage of porosity of the biomaterial. Garcia et al. (2021) [25] developed porcine collagen scaffolds with natural extracts containing or not containing hydroxyapatite for use in bone regeneration with a porosity of between 64.71 ± 7.11 and 96.23 ± 1.95%. The porosity percentage of the CCJ and CHER scaffolds (83.6 ± 3.1 and 86.7 ± 5.1%, respectively) was within the expected range for biomaterials used in bone regeneration, at 80 to 90% [80].

The absorption capacity of the biomaterial plays an important role for bone regeneration in terms of maintaining an adequate supply of nutrients for cell adhesion and proliferation [81]. Tsai et al. (2007) [82] obtained collagen/chitosan scaffolds with an absorption capacity of 135%. The CCJ and CHER scaffolds showed a higher absorption capacity of 2300% and 3470%, respectively. This high capacity to absorb liquids indicates that scaffolds can be applied in the area of tissue regeneration.

### 3.2. Clinical Analysis of Scaffold Grafting in the Repair of Mandibular Lesions in Rats

**Figure 7:**
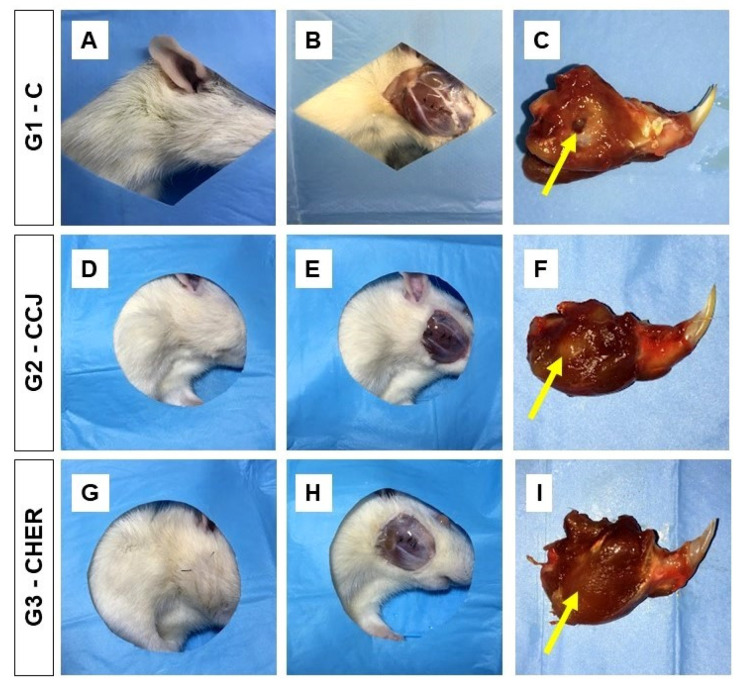
Macroscopic characteristics of the bone lesion in the ramus of the mandibles of rats in groups G1 (**A**–**C**), G2 (**D**–**F**), and G3 (**G**–**I**). There were no changes suggestive of infection in the surgical area. It was possible to see the membrane (yellow arrows) in some samples (**F**).

Clinical analysis of the surgical area of the mandibular branch of the animals used in this study showed normal hair growth, as well as skin coloration and healing, six weeks after surgery. In addition, the operated areas were lined with integral local musculature and adhered well to the mandible, with no clinical signs of complications such as infections, purulent secretion, tissue necrosis, or any other signs that would also indicate clinical signs of rejection of the scaffolds used in G2 and G3 (Figure 7). Therefore, the protocol used in the surgical procedure was within the expected standards as described in the experimental literature on mandibular regeneration [75].

**Figure 8:**
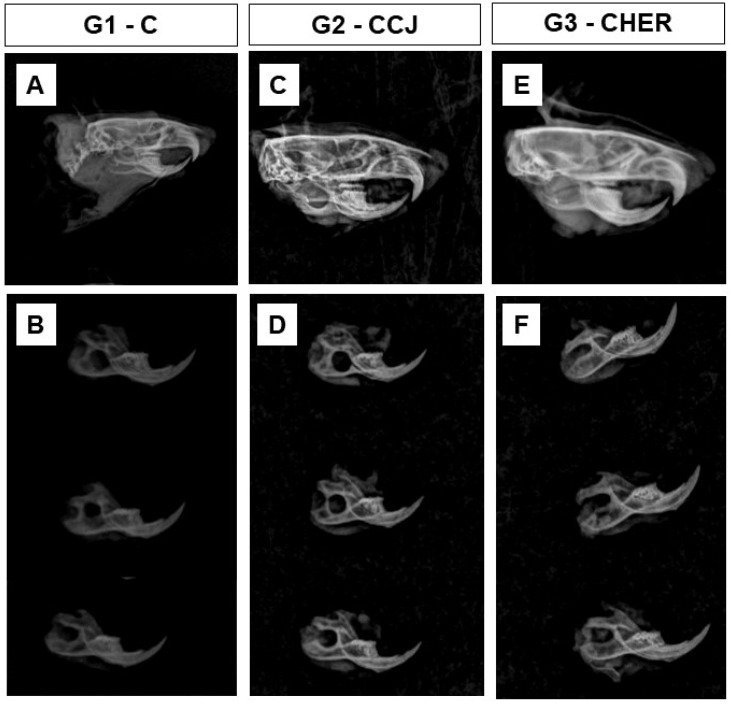
Radiological images of the skull and ramus of the mandibles of rats in groups G1 (**A**,**B**), G2 (**C**,**D**), and G3 (**E**,**F**). Note the integrity of the bone defect maintained 6 weeks after surgery and the good radiopacity of the mandible.

Radiographic images showed bone integrity adjacent to the lesion area, as there were no secondary fractures, bone rarefaction, or other signs indicating post-operative complications (Figure 8). In addition, the maxillomandibular occlusion of the animals was maintained and they ate normally throughout the experiment. With these data, the scaffolds used can be considered compatible with the host tissue of the mandible and are in line with the literature that proves the absence of toxicity given their chemical preparation by the alkaline hydrolysis method, which removes all the cellular components of the input used for their production [23,35,83]. In addition, the antioxidant and anti-inflammatory properties of the plants used in this research, which were jatoba and pomegranate, guarantee their indication in tissue-healing processes [28].

**Figure 9:**
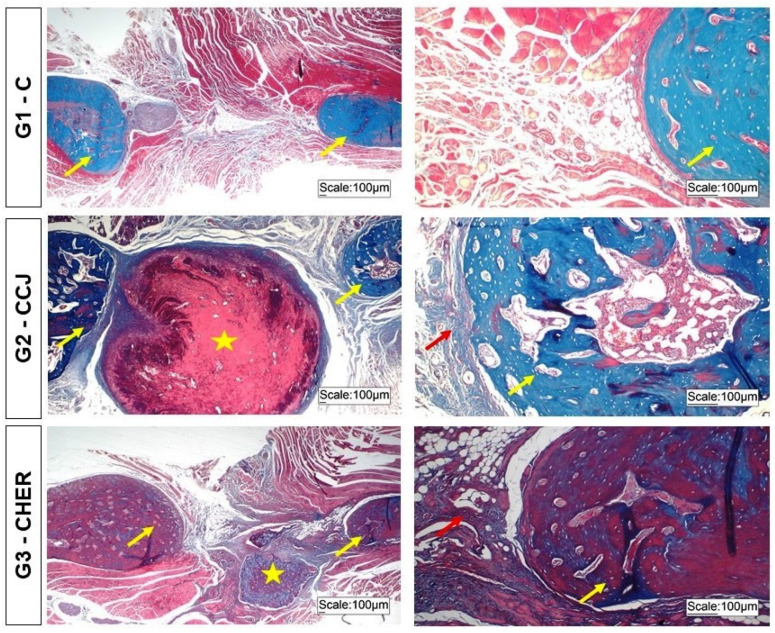
Histological images of the bone lesions in the branches of the mandibles of rats in groups G1 to G3 stained with Masson’s trichrome. Note the bone neoformation (yellow arrows) from the edges of the bone lesion and remnants of the scaffolds (star) implanted in G2 and G3. Reactive connective tissue (red arrows).

In the morphological analysis of this study, the initial concern was to identify any inflammatory infiltrate in the histological slides of the mandibular surgical area, especially in the rats in groups G2 and G3 that received the collagen/chitosan/jatoba and collagen/nanohydroxyapatite/elastin/roman scaffolds, respectively. Once the absence of any inflammatory exudate cells was confirmed, thus indicating the compatibility of these scaffolds (Figure 9), a discussion of the histomorphometric analysis of bone neoformation in the surgical area was then carried out in order to prove the osteogenic action of the standardized protocol.

In the surgical areas of the mandible of the animals in the control group, there was little formation of young bone due to the predominance of connective tissue occupying most of the defect area, as also described in other studies on mandible reconstruction but with different biomaterials [20,52]. In the areas implanted with the scaffolds used in this study, there was a slight increase in bone formation from the margins of the lesion compared to the control group, but this was still insufficient for total bone repair of the defect (Figure 9). Another characteristic observed in this study was the presence of remnants of the scaffolds, showing that they had not been completely degraded and bioresorbed. Thus, the biomaterial should not be absorbed quickly so that it can perform its support and osteoconductor function concomitantly during bone repair [54,84].

The histomorphometric analysis of this experimental protocol showed that the mean percentage of bone volume formed in the surgical area of groups G1, G2, and G3 was 17.17 ± 2.68, 27.45 ± 1.65, and 34.07 ± 0.64 (mean ± standard deviation), respectively. From the statistical analysis, all possible comparisons between the groups were significantly different (Table 1). Although the bone volume formed in both groups grafted with the scaffolds was greater than the control, it was still insufficient for complete bone healing within the experimental period of six weeks after the creation of the defect in the rats’ mandibles.

**Figure 10:**
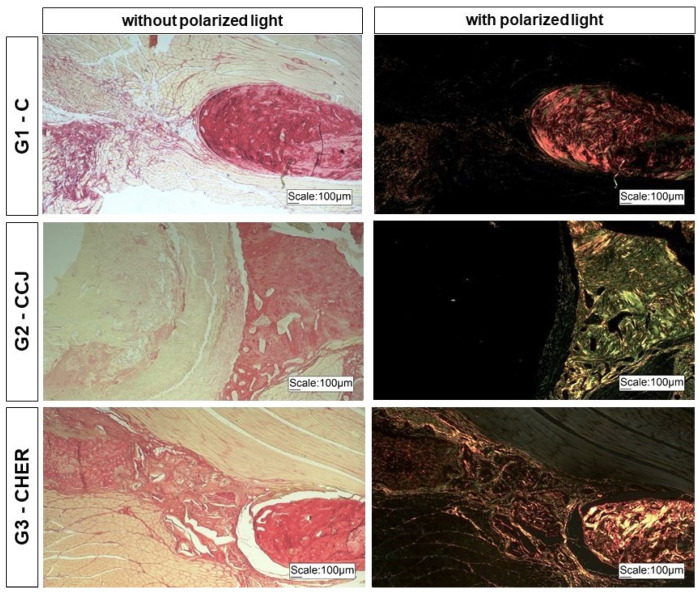
Photomicrographs of the bone lesions in the branches of the mandibles of rats in groups G1 to G3 stained with Picrosirius red in the absence and presence of polarized light. Note the birefringence of the tissues present in the bone lesion.

However, the osteogenic capacity of the scaffolds should be considered, given that there was greater bone formation than in the control group and the good birefringence of the bone and extracellular matrix in the area studied (Figure 10).

Zahedi et al. (1998) [52] also evaluated the repair of mandibular defects in rats but using a bovine collagen type I membrane cross-linked with diphenylphosphorylazide and found that after one month, the lesion had closed with new bone in most cases and that after 90 and 180 days, all the grafted experimental defects had completely regenerated. Meanwhile, Fan et al. (2014) [20] compared the use of adipose tissue-derived stem cells (ASCs) and bone morphogenetic protein-2 (BMP-2) in three-dimensional scaffolds composed of chitosan (CH) and chondroitin sulfate (CS) and with pore sizes ranging from 100 to 150 μm. In their results eight weeks after surgery in the mandible, they noted that in the group that received only the scaffold, the bone volume formed did not reach 20%, while the scaffolds together with ASC and BMP showed bone volume between 30 and 40%, according to the analysis of computerized microtomography. Even so, the authors concluded that the scaffolds used improved bone regeneration in rat mandibular defects.

In comparison, the volume of bone formation in the groups grafted with the scaffolds in this study was between 25% and 35%. Thus, both materials can be considered promising for tissue engineering due to the results obtained in the short term from the surgical powder established in this experiment and by comparing them with the bone volume values presented in the aforementioned literature with different biomaterials. Therefore, more studies are needed, as there are several factors that interfere with the healing process, such as experimental protocol, defect size, animal lineage, age, anatomical location, surgical technique, and postoperative evaluation period [20,85]. In addition, the properties of scaffolds also merit discussion, as there are still conflicts in the literature as to the ideal porosity to stimulate cell growth. In this research, there was an average pore size of 24 ± 7 µm for CCJ and 40 ± 17 µm for CHER.

Zhang et al. (2023) [86] state that bioceramic pores with a size above 200 μm are necessary for bone formation and vascularization. Research on hydroxyapatite reports that small pores of between 50 and 100 μm are used to induce endochondral ossification and larger pores of between 100 and 300 μm are for intramembranous ossification [87]. Garcia et al. (2021) [25] developed nanohydroxyapatite (nHA) and anionic collagen (C) scaffolds combined with plant extracts derived from grape seed, pomegranate peel, and jabuticaba peel and reported that they are promising biomaterials for osteogenesis due to their smaller pores (100 μm), which aid cell adhesion. Fan et al. (2014) [20] reported the osteogenic capacity of three-dimensional scaffolds composed of chitosan (CH) and chondroitin sulfate (CS) and with pore sizes ranging from 100 to 150 μm. Another report [61] described that the use of quince seed scaffolds with an interconnected porous network of around 233 ± 40 μm and 64 ± 15 μm provides nutrients necessary for bone regeneration. Therefore, the exact comparison of porosity analysis that can mimic an in vivo microenvironment for bone growth remains a challenge, as there are differences in porosity when comparing the same or similar scaffolds but also between different natural and synthetic materials.

In this context, it is necessary to standardize not only the three-dimensional porous structure of the scaffolds but also the animal model, the critical size of the experimental defects in the mandible, and the evaluation period, as these are factors that directly influence bone regeneration [20,88,89,90]. In this study, a period of six weeks was standardized after grafting with the scaffolds, and the porous structure may favor cell growth over a longer period. Therefore, we believe that the polymeric scaffold with pomegranate and jatoba used in this research has the capacity to stimulate bone growth, but over longer periods of tissue recovery. A limitation of this study is the fact that the short period of six weeks after surgery may not have been enough to completely close the bone defect filled with the scaffolds, despite the fact that the bone volume was greater than the control group in this study. Immunostainings can also complement histological findings. The promising results of scaffolds in the formation of new bone, their ease of production, and their low cost demonstrate the potential for translation from the bench to the bedside.

## 4. Conclusions

The combinatorial strategy of plant-derived extracts (pomegranate extract and jatoba resin) with polymeric scaffolds used in this research proved to be suitable for clinical use, with no foreign-body reaction and osteoconduction characteristics. In addition, these scaffolds can be considered viable products, as they are easily manipulated and have a low production cost, and, although the volume of newly formed bone was insufficient to repair the defect in the mandible in the short term, it was still superior to the control group. Therefore, further research with these scaffolds associated with phytochemical compounds from plants is important for tissue engineering, as it should also be considered that maxillofacial bones have a different embryological origin from other skeletal bones and consequently have a different regenerative capacity compared to grafts with biomaterials.

## Figures and Tables

**Table 1:** Percentage of bone volume formed in the surgical area.

G1-C	G2-CCJ	G3-CHER
17.17 ± 2.68 a	27.45 ± 1.65 b	34.07 ± 0.64 c

Line comparison, different lowercase letters indicate a significant difference, G1-C vs. G2-CCJ vs. G3-CHER (a ≠ b ≠ c). Mean ± standard deviation. ANOVA and Tukey’s post-test, *p* < 0.05.

## Data Availability

The data presented in this study are available on request from the corresponding author.
